# The Arrival of a New Car

**Published:** 1910-02-26

**Authors:** 


### Motoring Notes.

### THE ARRIVAL OF A NEW CAR.

The arrival of a new car is generally a long-expected and frequently much-delayed event, occasioning a thrill of excitement in the bosom of even the veteran motorist, which with the tyro is often accompanied by an amount of anxiety regarding the newcomer's constitution and treatment. It is unlikely in these days that an absolute novice will receive his car without an opportunity for practical instruction of some kind, as many did in the early days of the movement, and this should be followed—if not, as it should be, accompanied—by a careful study of the use and function of every part of the mechanism. Where the makers issue a handbook of their car—and most firms do nowadays—a perusal of this will be of material assistance. Where the owner is already familiar with some other type of car, special note should be taken of any important differences in the control arrangements, as the general tendency to more or less uniformity in these makes small discrepancies more puzzling. Special note should be taken of the position of every lubricator, grease cup, and other oiling arrangements.

The chief attention a new car requires is in the matter of lubrication, which should be free and plentiful, with due consideration for the troubles induced by excessive supply to the engine. This usually causes rapid fouling of ignition points; but a new motor generally lets more oil past the piston at first than after a little running, so the supply should not be cut down too much from this symptom unless persistent. All important nuts and bolts should be scrutinised for looseness after a run, especially those supporting detached parts of the mechanism, such as the silencer, carburettor, piping, etc., not, of course, omitting those of the steering-gear and brakes. It is not always superfluous to verify the freedom of petrol, water, and lubricating oil pipes from obstructions arising from dirt or waste left in the tanks.

The novice may probably find the following *résumé* of operations useful in the starting of his car, it being impossible, of course, to include variations necessary with some types. First fill the petrol tank, using a funnel with a strainer. Next fill the radiator, and see that the cock at the bottom of the water system is closed. Fill all grease boxes, if present; see that axle boxes are lubricated, and that the gear box and the differential case are plentifully supplied with lubricant. Fill all drip or other lubricators with a good motor oil—not too thick to travel along the pipes—and see that the crank case of the motor has its charge of the same, and that the steering is also attended to. The tyres should always be pumped up hard. It is presumed that the car has been registered, and that the necessary driving and Excise license has already been obtained.

In regard to the licenses, it may be useful to the embryo medical motorist to know that the driving license costing five shillings lasts for a year from the date on which it is taken out, and does not terminate on December 31, as the car license does, irrespective of the date on which it is issued. Owing to the fact that the Budget did not pass last year, licenses are being issued now under the old regulations, consequently medical men at present will not receive the advantage of the half-rate license fee promised them in the new regulations for motor-car taxation.

